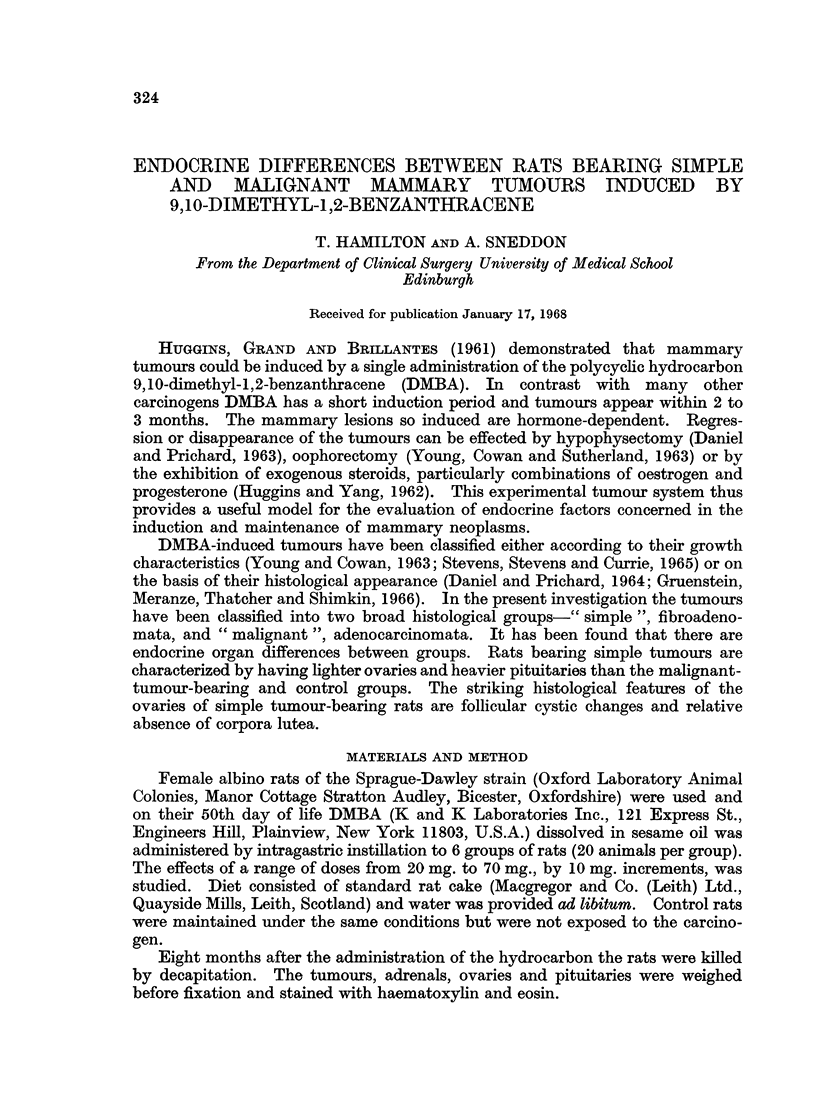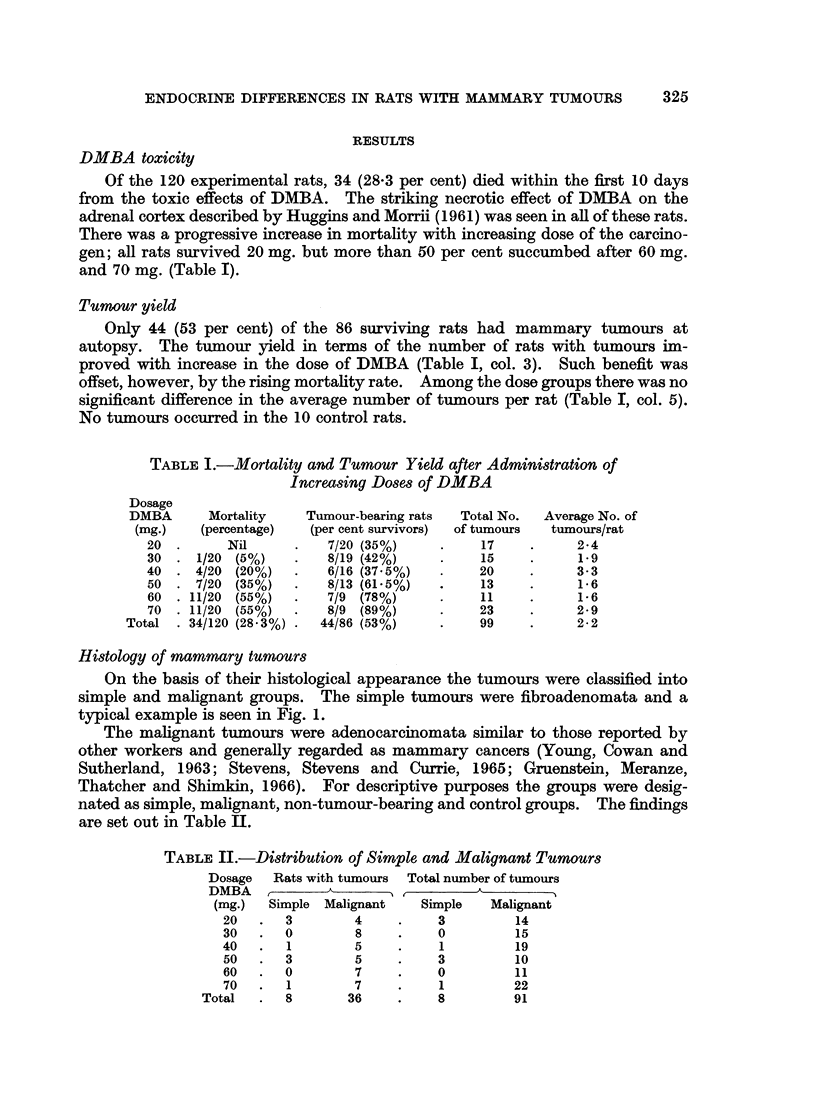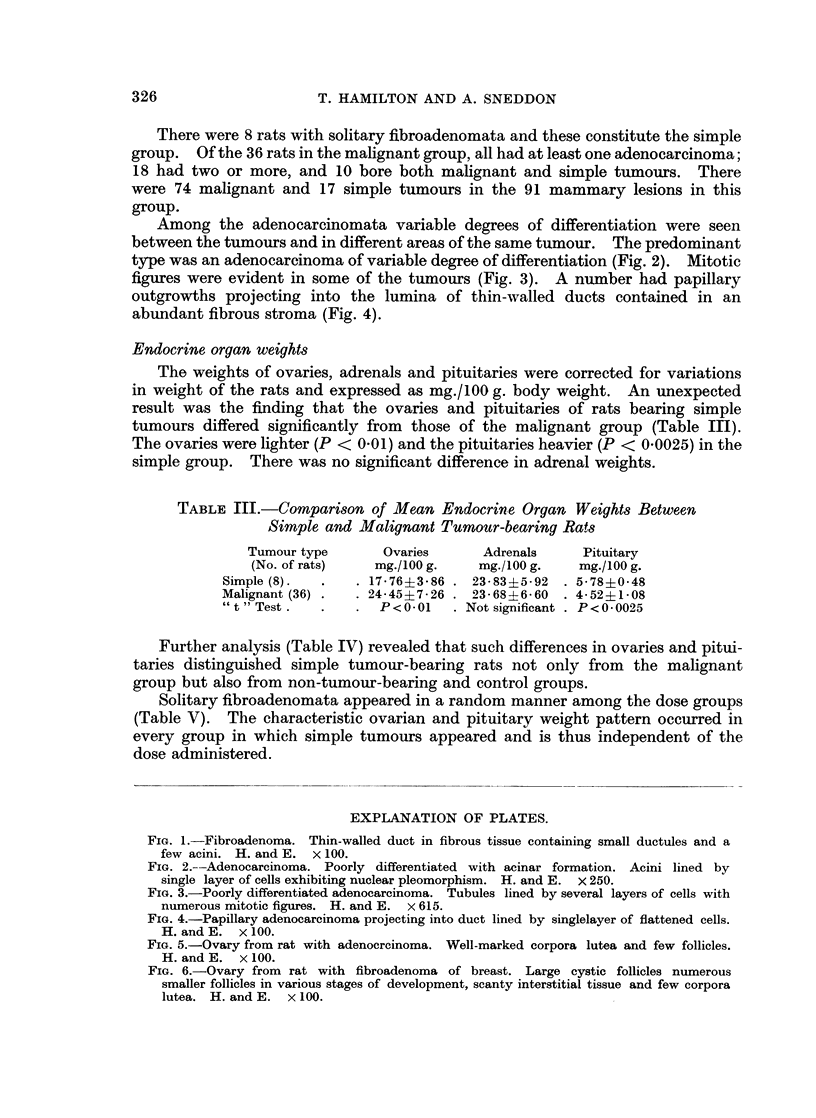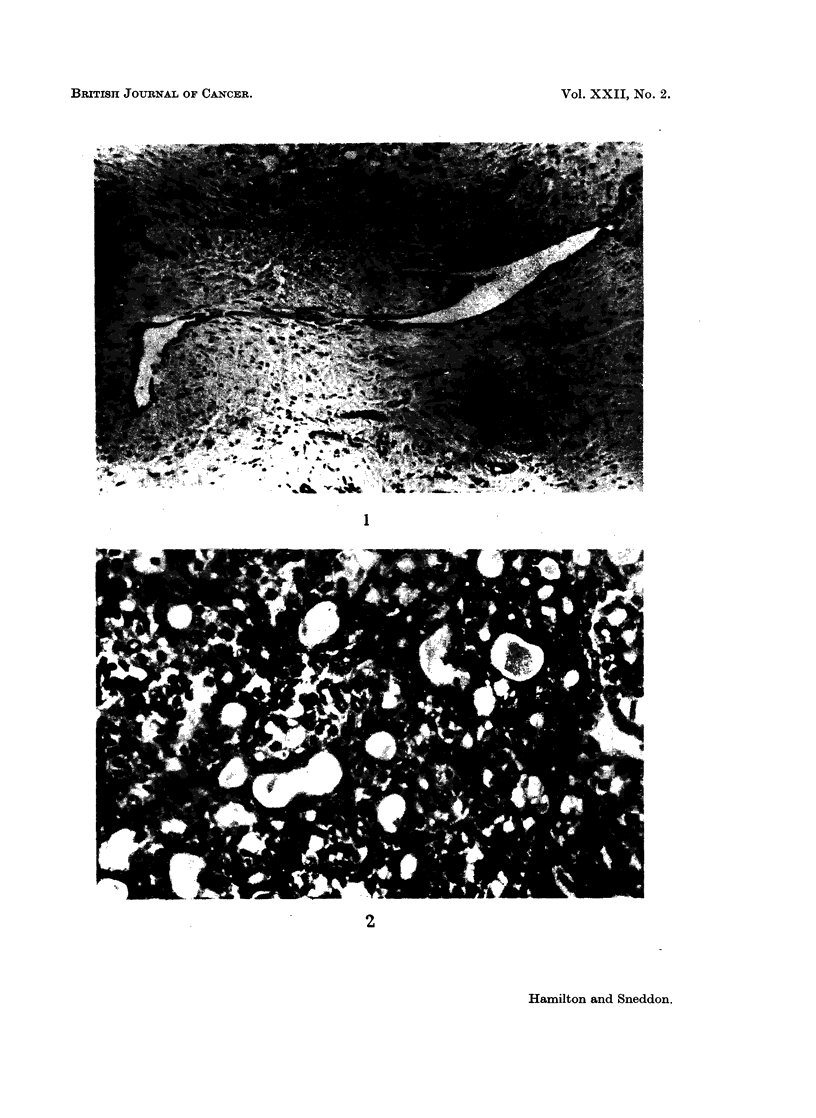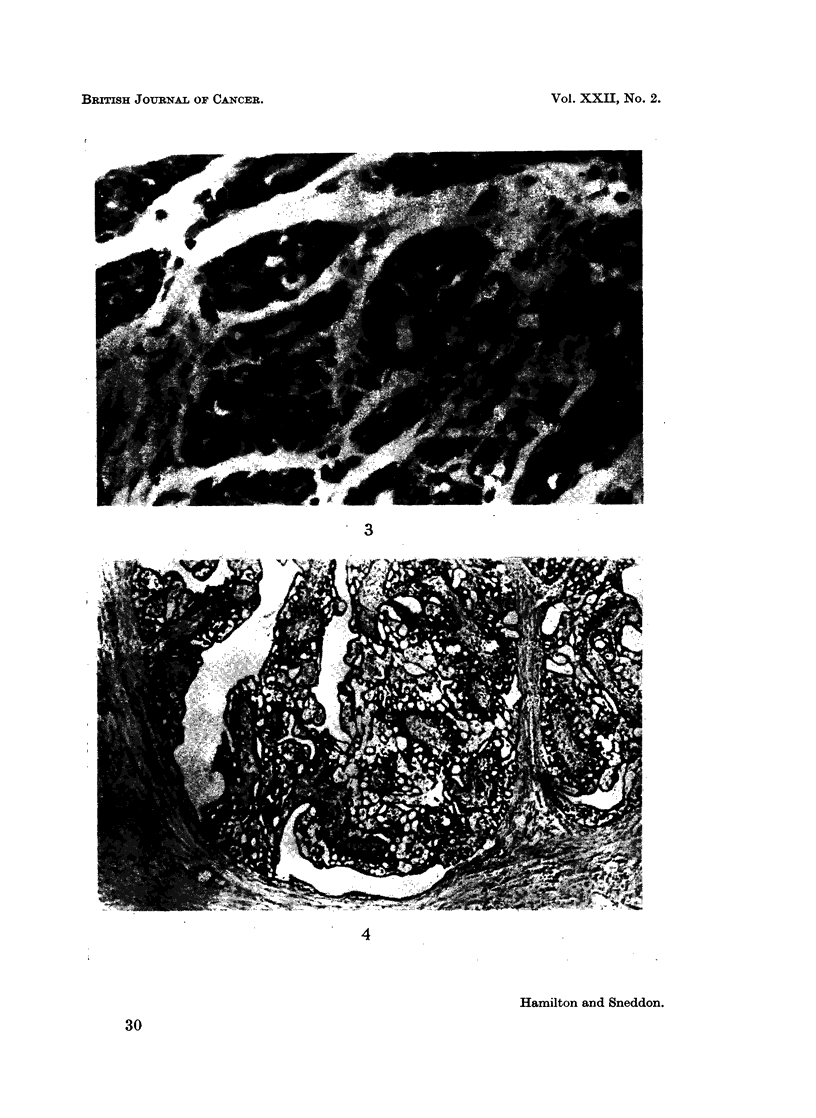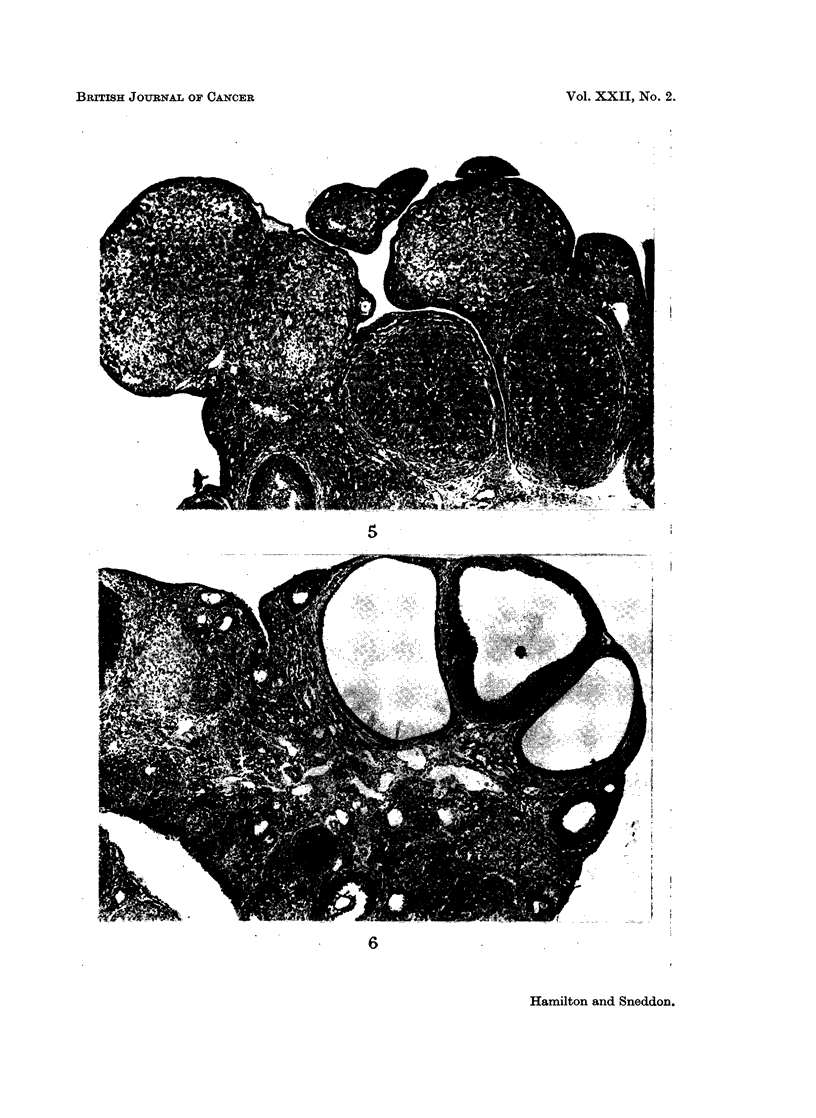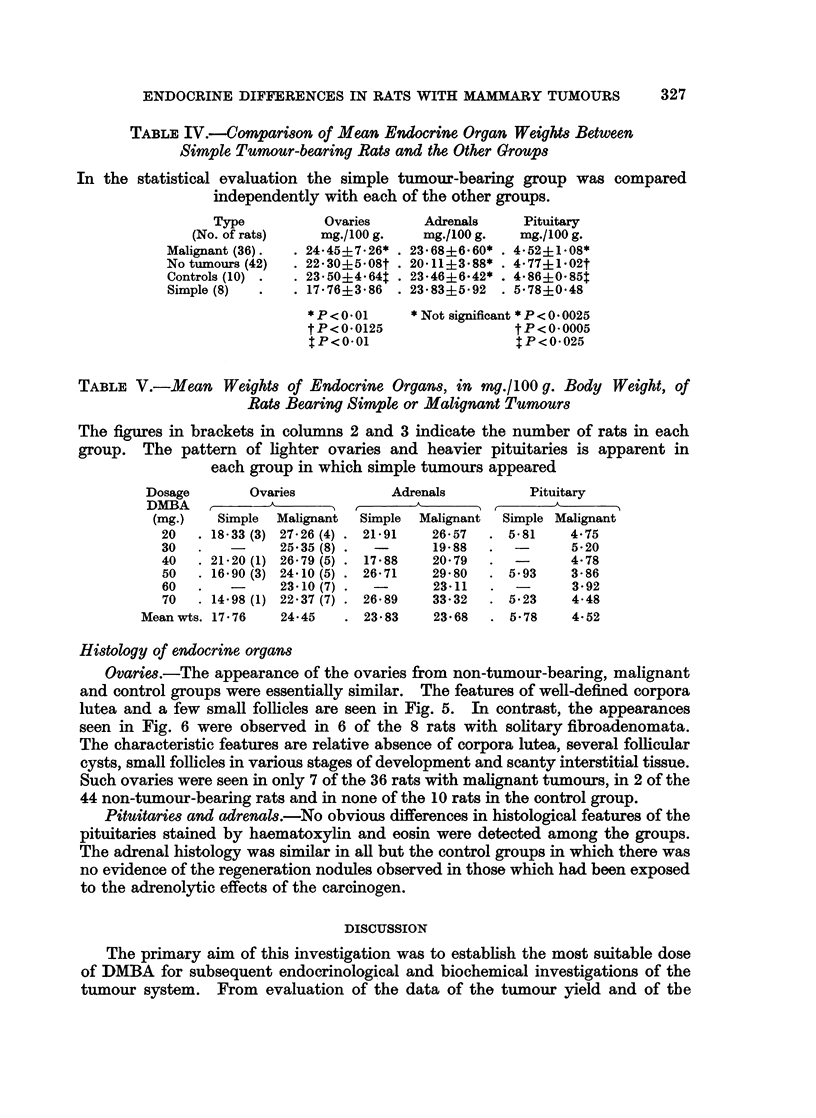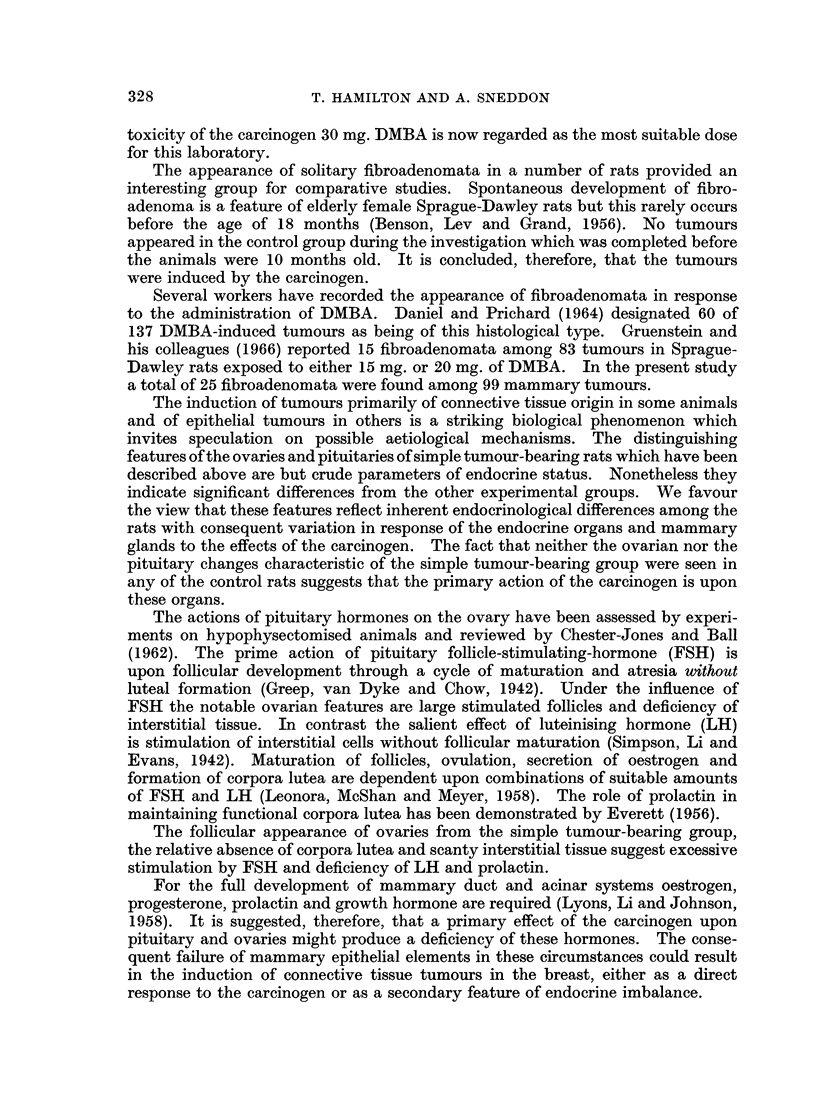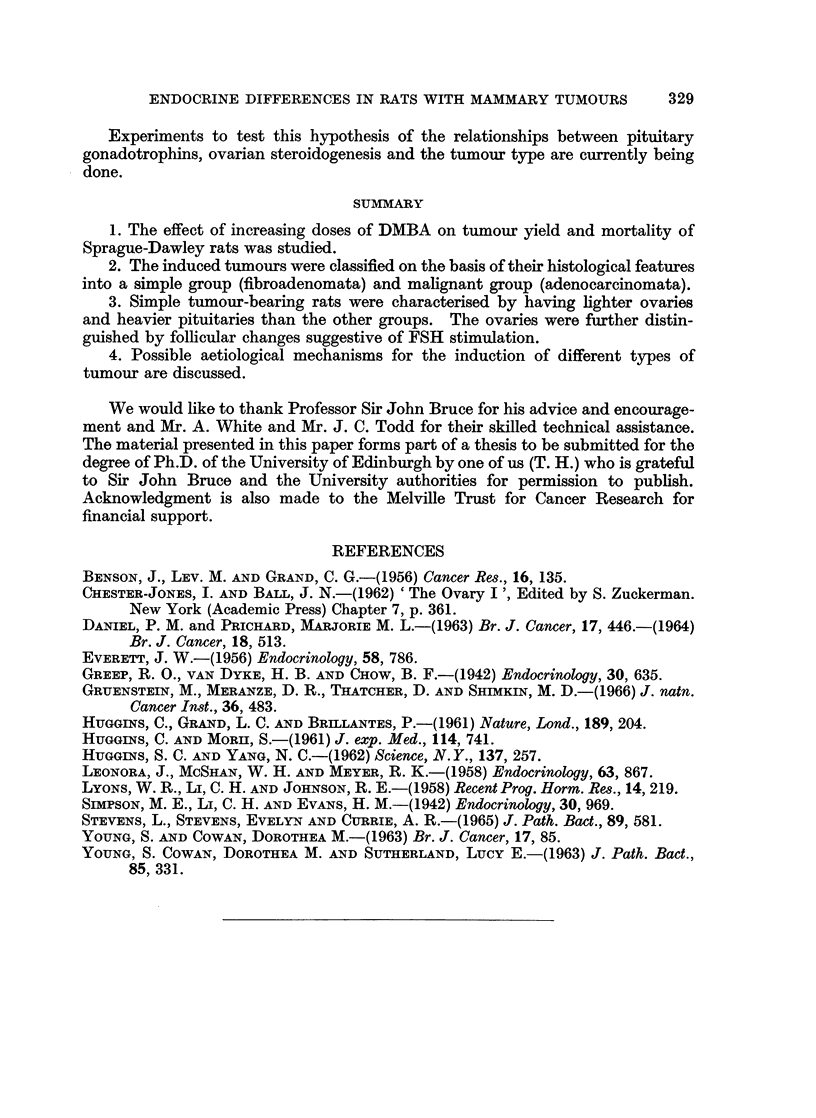# Endocrine differences between rats bearing simple and malignant mammary tumours induced by 9,10-dimethyl-1,2-benzanthracene.

**DOI:** 10.1038/bjc.1968.40

**Published:** 1968-06

**Authors:** T. Hamilton, A. Sneddon

## Abstract

**Images:**


### ENDOCRINE DIFFERENCES BETWEEN RATS BEARING SIMPLE AND MALIGNANT MAMMARY TUMOURS INDUCED BY 9,10-DIMETHYL-1,2-BENZANTHRACENE

T. HAMILTON AND A. SNEDDON

*From the Department of Clinical Surgery University of Medical School Edinburgh*

Received for publication January 17, 1968

HUGGINS, GRAND AND BRILLANTES (1961) demonstrated that mammary tumours could be induced by a single administration of the polycyclic hydrocarbon 9,10-dimethyl-1,2-benzanthracene (DMBA). In contrast with many other carcinogens DMBA has a short induction period and tumours appear within 2 to 3 months. The mammary lesions so induced are hormone-dependent. Regression or disappearance of the tumours can be effected by hypophysectomy (Daniel and Prichard, 1963), oophorectomy (Young, Cowan and Sutherland, 1963) or by the exhibition of exogenous steroids, particularly combinations of oestrogen and progesterone (Huggins and Yang, 1962). This experimental tumour system thus provides a useful model for the evaluation of endocrine factors concerned in the induction and maintenance of mammary neoplasms.

DMBA-induced tumours have been classified either according to their growth characteristics (Young and Cowan, 1963; Stevens, Stevens and Currie, 1965) or on the basis of their histological appearance (Daniel and Prichard, 1964; Gruenstein, Meranze, Thatcher and Shimkin, 1966). In the present investigation the tumours have been classified into two broad histological groups—" simple ", fibroadenomata, and " malignant ", adenocarcinomata. It has been found that there are endocrine organ differences between groups. Rats bearing simple tumours are characterized by having lighter ovaries and heavier pituitaries than the malignant-tumour-bearing and control groups. The striking histological features of the ovaries of simple tumour-bearing rats are follicular cystic changes and relative absence of corpora lutea.

### MATERIALS AND METHOD

Female albino rats of the Sprague-Dawley strain (Oxford Laboratory Animal Colonies, Manor Cottage Stratton Audley, Bicester, Oxfordshire) were used and on their 50th day of life DMBA (K and K Laboratories Inc., 121 Express St., Engineers Hill, Plainview, New York 11803, U.S.A.) dissolved in sesame oil was administered by intragastric instillation to 6 groups of rats (20 animals per group). The effects of a range of doses from 20 mg. to 70 mg., by 10 mg. increments, was studied. Diet consisted of standard rat cake (Macgregor and Co. (Leith) Ltd., Quayside Mills, Leith, Scotland) and water was provided *ad libitum*. Control rats were maintained under the same conditions but were not exposed to the carcinogen.

Eight months after the administration of the hydrocarbon the rats were killed by decapitation. The tumours, adrenals, ovaries and pituitaries were weighed before fixation and stained with haematoxylin and eosin.

### RESULTS

### *DMBA toxicity*

Of the 120 experimental rats, 34 (28·3 per cent) died within the first 10 days from the toxic effects of DMBA. The striking necrotic effect of DMBA on the adrenal cortex described by Huggins and Morrii (1961) was seen in all of these rats. There was a progressive increase in mortality with increasing dose of the carcinogen; all rats survived 20 mg. but more than 50 per cent succumbed after 60 mg. and 70 mg. (Table I).

### *Tumour yield*

Only 44 (53 per cent) of the 86 surviving rats had mammary tumours at autopsy. The tumour yield in terms of the number of rats with tumours improved with increase in the dose of DMBA (Table I, col. 3). Such benefit was offset, however, by the rising mortality rate. Among the dose groups there was no significant difference in the average number of tumours per rat (Table I, col. 5). No tumours occurred in the 10 control rats.

TABLE I.—*Mortality and Tumour Yield after Administration of Increasing Doses of DMBA*

| Dosage DMBA (mg.) | Mortality (percentage) | Tumour-bearing rats (per cent survivors) | Total No. of tumours | Average No. of tumours/rat |
|---|---|---|---|---|
| 20 | Nil | 7/20 (35%) | 17 | 2·4 |
| 30 | 1/20 (5%) | 8/19 (42%) | 15 | 1·9 |
| 40 | 4/20 (20%) | 6/16 (37·5%) | 20 | 3·3 |
| 50 | 7/20 (35%) | 8/13 (61·5%) | 13 | 1·6 |
| 60 | 11/20 (55%) | 7/9 (78%) | 11 | 1·6 |
| 70 | 11/20 (55%) | 8/9 (89%) | 23 | 2·9 |
| Total | 34/120 (28·3%) | 44/86 (53%) | 99 | 2·2 |

### *Histology of mammary tumours*

On the basis of their histological appearance the tumours were classified into simple and malignant groups. The simple tumours were fibroadenomata and a typical example is seen in Fig. 1.

The malignant tumours were adenocarcinomata similar to those reported by other workers and generally regarded as mammary cancers (Young, Cowan and Sutherland, 1963; Stevens, Stevens and Currie, 1965; Gruenstein, Meranze, Thatcher and Shimkin, 1966). For descriptive purposes the groups were designated as simple, malignant, non-tumour-bearing and control groups. The findings are set out in Table II.

TABLE II.—*Distribution of Simple and Malignant Tumours*

| Dosage DMBA (mg.) | Rats with tumours | | Total number of tumours | |
|---|---|---|---|---|
| | Simple | Malignant | Simple | Malignant |
| 20 | 3 | 4 | 3 | 14 |
| 30 | 0 | 8 | 0 | 15 |
| 40 | 1 | 5 | 1 | 19 |
| 50 | 3 | 5 | 3 | 10 |
| 60 | 0 | 7 | 0 | 11 |
| 70 | 1 | 7 | 1 | 22 |
| Total | 8 | 36 | 8 | 91 |

There were 8 rats with solitary fibroadenomata and these constitute the simple group. Of the 36 rats in the malignant group, all had at least one adenocarcinoma; 18 had two or more, and 10 bore both malignant and simple tumours. There were 74 malignant and 17 simple tumours in the 91 mammary lesions in this group.

Among the adenocarcinomata variable degrees of differentiation were seen between the tumours and in different areas of the same tumour. The predominant type was an adenocarcinoma of variable degree of differentiation (Fig. 2). Mitotic figures were evident in some of the tumours (Fig. 3). A number had papillary outgrowths projecting into the lumina of thin-walled ducts contained in an abundant fibrous stroma (Fig. 4).

*Endocrine organ weights*

The weights of ovaries, adrenals and pituitaries were corrected for variations in weight of the rats and expressed as mg./100 g. body weight. An unexpected result was the finding that the ovaries and pituitaries of rats bearing simple tumours differed significantly from those of the malignant group (Table III). The ovaries were lighter ($P < 0{\cdot}01$) and the pituitaries heavier ($P < 0{\cdot}0025$) in the simple group. There was no significant difference in adrenal weights.

TABLE III.—*Comparison of Mean Endocrine Organ Weights Between Simple and Malignant Tumour-bearing Rats*

| Tumour type (No. of rats) | Ovaries mg./100 g. | Adrenals mg./100 g. | Pituitary mg./100 g. |
|---|---|---|---|
| Simple (8) | $17{\cdot}76 \pm 3{\cdot}86$ | $23{\cdot}83 \pm 5{\cdot}92$ | $5{\cdot}78 \pm 0{\cdot}48$ |
| Malignant (36) | $24{\cdot}45 \pm 7{\cdot}26$ | $23{\cdot}68 \pm 6{\cdot}60$ | $4{\cdot}52 \pm 1{\cdot}08$ |
| "t" Test | $P < 0{\cdot}01$ | Not significant | $P < 0{\cdot}0025$ |

Further analysis (Table IV) revealed that such differences in ovaries and pituitaries distinguished simple tumour-bearing rats not only from the malignant group but also from non-tumour-bearing and control groups.

Solitary fibroadenomata appeared in a random manner among the dose groups (Table V). The characteristic ovarian and pituitary weight pattern occurred in every group in which simple tumours appeared and is thus independent of the dose administered.

---

EXPLANATION OF PLATES.

FIG. 1.—Fibroadenoma. Thin-walled duct in fibrous tissue containing small ductules and a few acini. H. and E. ×100.

FIG. 2.—Adenocarcinoma. Poorly differentiated with acinar formation. Acini lined by single layer of cells exhibiting nuclear pleomorphism. H. and E. ×250.

FIG. 3.—Poorly differentiated adenocarcinoma. Tubules lined by several layers of cells with numerous mitotic figures. H. and E. ×615.

FIG. 4.—Papillary adenocarcinoma projecting into duct lined by singlelayer of flattened cells. H. and E. ×100.

FIG. 5.—Ovary from rat with adenocrcinoma. Well-marked corpora lutea and few follicles. H. and E. ×100.

FIG. 6.—Ovary from rat with fibroadenoma of breast. Large cystic follicles numerous smaller follicles in various stages of development, scanty interstitial tissue and few corpora lutea. H. and E. ×100.

1

2

3

4

Hamilton and Sneddon.

5

6

TABLE IV.—*Comparison of Mean Endocrine Organ Weights Between Simple Tumour-bearing Rats and the Other Groups*

In the statistical evaluation the simple tumour-bearing group was compared independently with each of the other groups.

| Type (No. of rats) | Ovaries mg./100 g. | Adrenals mg./100 g. | Pituitary mg./100 g. |
|---|---|---|---|
| Malignant (36) | 24·45±7·26* | 23·68±6·60* | 4·52±1·08* |
| No tumours (42) | 22·30±5·08† | 20·11±3·88* | 4·77±1·02† |
| Controls (10) | 23·50±4·64‡ | 23·46±6·42* | 4·86±0·85‡ |
| Simple (8) | 17·76±3·86 | 23·83±5·92 | 5·78±0·48 |
| | * $P<0·01$<br>† $P<0·0125$<br>‡ $P<0·01$ | * Not significant | * $P<0·0025$<br>† $P<0·0005$<br>‡ $P<0·025$ |

TABLE V.—*Mean Weights of Endocrine Organs, in mg./100 g. Body Weight, of Rats Bearing Simple or Malignant Tumours*

The figures in brackets in columns 2 and 3 indicate the number of rats in each group. The pattern of lighter ovaries and heavier pituitaries is apparent in each group in which simple tumours appeared

| Dosage DMBA (mg.) | Ovaries | | Adrenals | | Pituitary | |
|---|---|---|---|---|---|---|
| | Simple | Malignant | Simple | Malignant | Simple | Malignant |
| 20 | 18·33 (3) | 27·26 (4) | 21·91 | 26·57 | 5·81 | 4·75 |
| 30 | — | 25·35 (8) | — | 19·88 | — | 5·20 |
| 40 | 21·20 (1) | 26·79 (5) | 17·88 | 20·79 | — | 4·78 |
| 50 | 16·90 (3) | 24·10 (5) | 26·71 | 29·80 | 5·93 | 3·86 |
| 60 | — | 23·10 (7) | — | 23·11 | — | 3·92 |
| 70 | 14·98 (1) | 22·37 (7) | 26·89 | 33·32 | 5·23 | 4·48 |
| Mean wts. | 17·76 | 24·45 | 23·83 | 23·68 | 5·78 | 4·52 |

*Histology of endocrine organs*

*Ovaries.*—The appearance of the ovaries from non-tumour-bearing, malignant and control groups were essentially similar. The features of well-defined corpora lutea and a few small follicles are seen in Fig. 5. In contrast, the appearances seen in Fig. 6 were observed in 6 of the 8 rats with solitary fibroadenomata. The characteristic features are relative absence of corpora lutea, several follicular cysts, small follicles in various stages of development and scanty interstitial tissue. Such ovaries were seen in only 7 of the 36 rats with malignant tumours, in 2 of the 44 non-tumour-bearing rats and in none of the 10 rats in the control group.

*Pituitaries and adrenals.*—No obvious differences in histological features of the pituitaries stained by haematoxylin and eosin were detected among the groups. The adrenal histology was similar in all but the control groups in which there was no evidence of the regeneration nodules observed in those which had been exposed to the adrenolytic effects of the carcinogen.

DISCUSSION

The primary aim of this investigation was to establish the most suitable dose of DMBA for subsequent endocrinological and biochemical investigations of the tumour system. From evaluation of the data of the tumour yield and of the toxicity of the carcinogen 30 mg. DMBA is now regarded as the most suitable dose for this laboratory.

The appearance of solitary fibroadenomata in a number of rats provided an interesting group for comparative studies. Spontaneous development of fibroadenoma is a feature of elderly female Sprague-Dawley rats but this rarely occurs before the age of 18 months (Benson, Lev and Grand, 1956). No tumours appeared in the control group during the investigation which was completed before the animals were 10 months old. It is concluded, therefore, that the tumours were induced by the carcinogen.

Several workers have recorded the appearance of fibroadenomata in response to the administration of DMBA. Daniel and Prichard (1964) designated 60 of 137 DMBA-induced tumours as being of this histological type. Gruenstein and his colleagues (1966) reported 15 fibroadenomata among 83 tumours in Sprague-Dawley rats exposed to either 15 mg. or 20 mg. of DMBA. In the present study a total of 25 fibroadenomata were found among 99 mammary tumours.

The induction of tumours primarily of connective tissue origin in some animals and of epithelial tumours in others is a striking biological phenomenon which invites speculation on possible aetiological mechanisms. The distinguishing features of the ovaries and pituitaries of simple tumour-bearing rats which have been described above are but crude parameters of endocrine status. Nonetheless they indicate significant differences from the other experimental groups. We favour the view that these features reflect inherent endocrinological differences among the rats with consequent variation in response of the endocrine organs and mammary glands to the effects of the carcinogen. The fact that neither the ovarian nor the pituitary changes characteristic of the simple tumour-bearing group were seen in any of the control rats suggests that the primary action of the carcinogen is upon these organs.

The actions of pituitary hormones on the ovary have been assessed by experiments on hypophysectomised animals and reviewed by Chester-Jones and Ball (1962). The prime action of pituitary follicle-stimulating-hormone (FSH) is upon follicular development through a cycle of maturation and atresia *without* luteal formation (Greep, van Dyke and Chow, 1942). Under the influence of FSH the notable ovarian features are large stimulated follicles and deficiency of interstitial tissue. In contrast the salient effect of luteinising hormone (LH) is stimulation of interstitial cells without follicular maturation (Simpson, Li and Evans, 1942). Maturation of follicles, ovulation, secretion of oestrogen and formation of corpora lutea are dependent upon combinations of suitable amounts of FSH and LH (Leonora, McShan and Meyer, 1958). The role of prolactin in maintaining functional corpora lutea has been demonstrated by Everett (1956).

The follicular appearance of ovaries from the simple tumour-bearing group, the relative absence of corpora lutea and scanty interstitial tissue suggest excessive stimulation by FSH and deficiency of LH and prolactin.

For the full development of mammary duct and acinar systems oestrogen, progesterone, prolactin and growth hormone are required (Lyons, Li and Johnson, 1958). It is suggested, therefore, that a primary effect of the carcinogen upon pituitary and ovaries might produce a deficiency of these hormones. The consequent failure of mammary epithelial elements in these circumstances could result in the induction of connective tissue tumours in the breast, either as a direct response to the carcinogen or as a secondary feature of endocrine imbalance.

Experiments to test this hypothesis of the relationships between pituitary gonadotrophins, ovarian steroidogenesis and the tumour type are currently being done.

### SUMMARY

1. The effect of increasing doses of DMBA on tumour yield and mortality of Sprague-Dawley rats was studied.

2. The induced tumours were classified on the basis of their histological features into a simple group (fibroadenomata) and malignant group (adenocarcinomata).

3. Simple tumour-bearing rats were characterised by having lighter ovaries and heavier pituitaries than the other groups. The ovaries were further distinguished by follicular changes suggestive of FSH stimulation.

4. Possible aetiological mechanisms for the induction of different types of tumour are discussed.

We would like to thank Professor Sir John Bruce for his advice and encouragement and Mr. A. White and Mr. J. C. Todd for their skilled technical assistance. The material presented in this paper forms part of a thesis to be submitted for the degree of Ph.D. of the University of Edinburgh by one of us (T. H.) who is grateful to Sir John Bruce and the University authorities for permission to publish. Acknowledgment is also made to the Melville Trust for Cancer Research for financial support.

### REFERENCES

Benson, J., Lev. M. and Grand, C. G.—(1956) *Cancer Res.*, **16**, 135.

Chester-Jones, I. and Ball, J. N.—(1962) ‘ The Ovary I ’, Edited by S. Zuckerman. New York (Academic Press) Chapter 7, p. 361.

Daniel, P. M. and Prichard, Marjorie M. L.—(1963) *Br. J. Cancer*, **17**, 446.—(1964) *Br. J. Cancer*, **18**, 513.

Everett, J. W.—(1956) *Endocrinology*, **58**, 786.

Greep, R. O., van Dyke, H. B. and Chow, B. F.—(1942) *Endocrinology*, **30**, 635.

Gruenstein, M., Meranze, D. R., Thatcher, D. and Shimkin, M. D.—(1966) *J. natn. Cancer Inst.*, **36**, 483.

Huggins, C., Grand, L. C. and Brillantes, P.—(1961) *Nature, Lond.*, **189**, 204.

Huggins, C. and Morii, S.—(1961) *J. exp. Med.*, **114**, 741.

Huggins, S. C. and Yang, N. C.—(1962) *Science, N.Y.*, **137**, 257.

Leonora, J., McShan, W. H. and Meyer, R. K.—(1958) *Endocrinology*, **63**, 867.

Lyons, W. R., Li, C. H. and Johnson, R. E.—(1958) *Recent Prog. Horm. Res.*, **14**, 219.

Simpson, M. E., Li, C. H. and Evans, H. M.—(1942) *Endocrinology*, **30**, 969.

Stevens, L., Stevens, Evelyn and Currie, A. R.—(1965) *J. Path. Bact.*, **89**, 581.

Young, S. and Cowan, Dorothea M.—(1963) *Br. J. Cancer*, **17**, 85.

Young, S. Cowan, Dorothea M. and Sutherland, Lucy E.—(1963) *J. Path. Bact.*, **85**, 331.